# A Broadband THz-TDS System Based on DSTMS Emitter and LTG InGaAs/InAlAs Photoconductive Antenna Detector

**DOI:** 10.1038/srep26949

**Published:** 2016-05-31

**Authors:** Ying Zhang, Xiaoling Zhang, Shaoxian Li, Jianqiang Gu, Yanfeng Li, Zhen Tian, Chunmei Ouyang, Mingxia He, Jiaguang Han, Weili Zhang

**Affiliations:** 1 Center for Terahertz Waves and College of Precision Instrument and Optoelectronics Engineering, Tianjin University, and Key Laboratory of Optoelectronics Information and Technology, Ministry of Education of China, Tianjin 300072, People’s Republic of China; 2 School of Electrical and Computer Engineering, Oklahoma State University, Stillwater, Oklahoma 74078, USA

## Abstract

We demonstrate a 4-f terahertz time-domain spectroscopy (THz-TDS) system using an organic crystal DSTMS as the THz emitter and a low temperature grown (LTG) InGaAs/InAlAs photoconductive antenna as the receiver. The system covers a frequency range from 0.2 up to 8 THz. The influences of the pump laser power, the probe laser power and the azimuthal angle of the DSTMS crystal on the time-domain THz amplitude are experimentally analyzed. The frequency accuracy of the system is verified by measuring two metamaterial samples and a lactose film in this THz-TDS system. The proposed combination of DSTMS emission and PC antenna detection realizes a compact and low-cost THz-TDS scheme with an ultra-broad bandwidth, which may promote the development and the applications of THz-TDS techniques.

Terahertz (THz) radiation covers a frequency range from 0.1–10 THz, which has many unique features such as low photon energy, good penetrability, and excellent capability for spectral analysis^1^,^2^. These make THz technology useful in different fields, including high-speed communication, non-destructive inspection, security check, biology research, gas sensing, cancer diagnosis and so on^3^,^4^,^5^. Among various THz techniques, THz time-domain spectroscopy (THz-TDS) is the most frequently used, in which material characteristics are recorded as time-domain THz electrical field pulses transmitted through or reflected from an object. Compared with other far-infrared spectroscopic techniques, THz-TDS with coherent detection has tremendous advantages in terms of high sensitivity, pure polarization and direct measurement of both real and imaginary parts of the dielectric function^6^. As a popular spectroscopy technique, bandwidth is one of the most important parameters for THz-TDS. A broader bandwidth enables a more accurate material identification ability. For example, particular sensitive chemicals such as TNT, RDX, HMX and 2, 4-DNT all have multi-signatures beyond 4 THz. However, the broadband THz-TDS schemes with over 6 THz bandwidth are usually very expensive and difficult to operate thus hindering their practical applications. One possible and easy solution here is to develop best matched THz sources and detectors thus bringing down the cost but remaining the broadband feature. Among different types of exist THz emitters and detectors, photoconductive (PC) switching and optical rectification (OR) have been widely used to emit picosecond THz pulses, and PC sampling and electro-optical (EO) sampling have been the two main THz wave coherent detection methods^7^,^8^,^9^,^10^. Recently, thanks to the development of organic crystals such as HMQ-TMS^11^, DAST^12^, DSTMS^13^,^14^ and OH1^15^, OR emission and EO detection can give a higher center frequency performance than PC antennas when the pump laser pulse width is longer than 50 fs. For example, the frequency range of most low temperature grown (LTG) GaAs PC antenna-based THz-TDS is limited by about 5 THz and the center frequency is less than 1 THz. Though ultra-broadband THz radiation up to 20 and 30 THz were obtained from semi-insulating and LTG GaAs PC antennas respectively^16^,^17^, the pump laser pulse width needed to be less than 15 fs. On the other hand, THz pulses with a center frequency higher than 1 THz can easily be obtained in nonlinear crystals such as ZnTe and GaP, even pumped by femtosecond lasers with a duration of tens of femtoseconds. By use of ultrashort laser pulses the generation of THz radiation by OR process has been reported to reach over 41 THz^18^.

Besides the consideration on the center frequency and the bandwidth, in recent years, more and more potential applications require optical-fiber-friendly THz-TDS systems pumped at 1550 nm, where LTG InGaAs/InAlAs based PC antennas and organic crystals such as DAST, DSTMS are used as promising THz sources^19^,^20^,^21^ and receivers^22^,^23^. The band coverage of LTG InGaAs/InAlAs based PC antennas can reach up to 5 THz but the center frequency is mainly less than 0.8 THz^24^,^25^,^26^. In contrast, the center frequency and the bandwidth of DSTMS emitters and detectors are much better. DSTMS has good velocity match at a wide range of wavelengths (from 720–1650 nm)^19^,^27^,^28^,^29^. When pumped with IR radiation between 1.3 and 1.5 μm, the radiated THz wave can fill the entire THz gap and the center frequency is up to 2 THz^30^. However, the PC antenna is much easier to integrate with the fiber technique, especially at the detection side, where as the optics of EO sampling is nearly impossible to be directly coupled with a fiber. Thus to realize an optical fiber coupled THz-TDS with a broad bandwidth and a high center frequency, a combination of DSTMS OR emission and LTG InGaAs/InAlAs PC sampling is strongly suggested. So far, this combination is still lack of experimental verification.

In this letter, we demonstrate a typical free-space 4 f THz-TDS system with a DSTMS crystal as the emitter and a LTG InGaAs/InAlAs PC antenna as the detector, which has a frequency coverage up to 8 THz with a center frequency at 1.44 THz. To verify the frequency accuracy of the proposed system, two different metamaterial samples: U-shape split-ring resonators (SRRs) and rectangle hole arrays, as well as a lactose film are tested in the system. The proposed DSTMS along with PC antenna scheme not only realizes a compact and low-cost ultra-broadband THz-TDS, but also inherits the fiber coupling capability of the PC antenna. The demonstrated system here may pave a new way for the development of ultra-broadband THz systems, thus promoting the applications of THz-TDS techniques in security check, drug prevention and medical research.

## Experimental setup

Since the first discovery of DAST, it has been used as a broadband and high-energy THz source. The organic crystal DSTMS is derived from DAST but has a lower absorption around 1 THz and a faster crystal growth rate^13^,^31^,^32^. What’s more, DSTMS has the best performances when pumped in the IR regime. For example, DSTMS has a nonlinear coefficient as high as 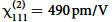 when pumped with IR radiation between 1.3 and 1.5 μm, as well as a good phase match. Therefore, DSTMS has become a favored potential emitter for assembling all fiber THz-TDS system and has been widely studied. In this work, a commercial DSTMS crystal from Rainbow Photonics is utilized as the THz emitter, which has a total area of 4 × 5 mm^2^ with a thickness of 0.3 mm. Although DSTMS has been utilized as detector in several previous works, its giant birefringence and EO sampling based detection optics hinder its utilization as a fiber-coupled THz receiver. Thus the detection of THz pulses in this work was accomplished by a commercial LTG InGaAs/InAlAs PC antenna (TERA15-DP25) from Menlo Systems GmbH. This is a typical H-type dipole antenna, which has a 4 × 4 mm^2^ total area and a 0.35 mm thickness with a 25 μm long dipole arm and a 10 μm wide PC gap. Compared with bare LTG InGaAs grown lattice-matched to InP substrate, beryllium-doped LTG InGaAs/InAlAs has a higher resistivity up to 10^6^ Ω/sq, a suppressed dark conductivity and a reduced relaxation time^33^,^34^. A 4.5 THz response bandwidth and a 60 dB dynamic range are specified by the manufacturer.

**Figure 1:**
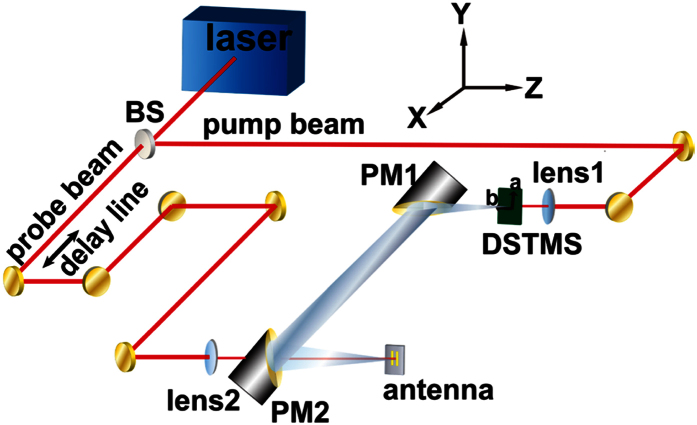
Schematic of the experimental setup. BS, beam splitter; PM, parabolic mirror.

The schematic of the DSTMS emission and LTG InGaAs/InAlAs PC antenna detection based THz-TDS system is shown in Fig. 1. The source is a 100 fs fiber amplifier (TOPTICA, FemtoFiber Pro NIR) operating at 1560 nm with an 80 MHz repetition rate. The output is split into two beams by using a beam splitter consisting of a λ/2 wave plate and a cubic polarizer, and the powers in each beam can be tuned by rotating the wave plate. One beam is used to pump the DSTMS crystal and the other one with an average power of 25 mW is used as the probe beam of the PC antenna. In front of the emitter there is a lens with a 50 mm focal length to adjust the beam size. The pump power measured in front of the crystal is 172.9 mW, which is the net average power used to pump the DSTMS crystal. The THz wave emitted from the DSTMS crystal is collimated and re-focused by two parabolic mirrors with a 50.8 mm effective focal length. A high-resistivity silicon wafer with a thickness of 500 μm is placed in the middle of the parabolic mirrors to block part of the residual pump beam. For concentrating the THz wave and shrinking the light spot, and at the same time, improving the signal to noise ratio (SNR), the crystal and the antenna are positioned at the respective foci of the parabolic mirrors. The probe beam with a power of 25 mW is focused onto the front side of the antenna by a lens with a focal length of 100 mm. Such an experimental scheme excludes the influences from the InP substrate and the silicon hyper-semispherical lens. The probe beam and THz spot overlap at the gap of the PC antenna and the THz signal is sampled by the photocurrent versus time delay. The amplitude and phase spectra are obtained by Fourier Transform of the temporal waveform of the detected THz pulses. The scanning time window of the waveform is set to 14 ps for cutting off the Fresnel reflection echo of the antenna substrate.

## Results and Discussion

**Figure 2:**
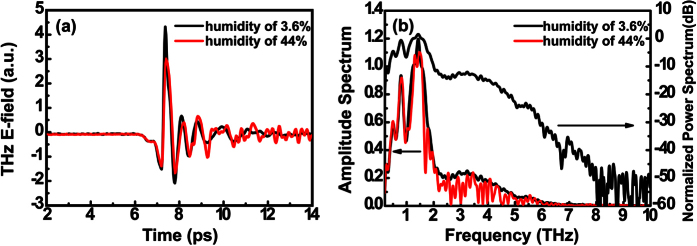
THz time-domain signal and its amplitude/power spectrum. (**a**) Time-domain traces of THz wave generated by DSTMS and detected by LTG InGaAS/InAlAs PC antenna. (**b**) Frequency spectra of THz wave calculated by Fourier transform. The red lines represent signals measured in air with a humidity of 44% and dark lines are those obtained in dry air environment.

Figure 2(a) shows the THz signals in the time domain measured under conditions of different humidity. A humidity of 44% causes a decrease in the THz amplitude and strong oscillations after the main peak. As the humidity is reduced to 3.6%, this disorder decreases and the peak amplitude of the THz pulse increases by 42.6%. Figure 2(b) shows the frequency spectra and the power spectrum calculated by Fourier transform, which shows an unexpected frequency coverage up to 8 THz and 4.4 THz (full width at 1/10 of maximum) bandwidth with a center frequency at 1.44 THz. The dynamic range obtained from the power spectrum in Fig. 2(b) is larger than 50 dB. This ultra-broad bandwidth, as far as we know, is far beyond the usually reported frequency range of 5 THz observed in InGaAs/InAlAs PC antenna. In the spectra, there are obvious absorption peaks around 1.15, 1.31, 1.42, 1.69, 1.81 THz due to ambient water vapor, which agrees with the values reported in other references^35^,^36^. There are two spectral valleys centered at 0.61 and 0.98 THz even in a dry-air environment. This is mainly caused by the characteristic absorption in the DSTMS crystal^13^,^37^, which is responsible for the time-domain oscillations following the main peak of the signal. However, in the spectrum there is a relatively broad dip around 2.6 THz that needs subsequent investigation in the future work to clarify its origin.

**Figure 3:**
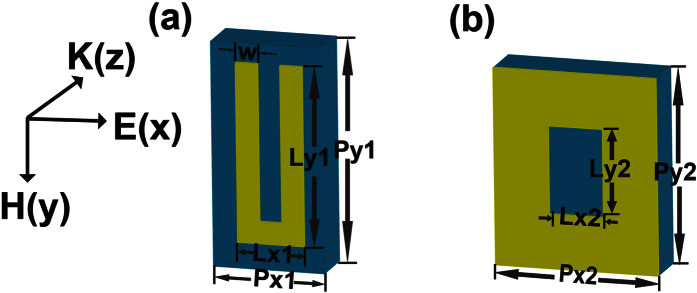
Unit Cell schematic of the metamaterial samples. (**a**) Schematic diagram of the U-shape SRR with geometrical parameters. Px1 = 25 μm, Py1 = 50 μm, Lx1 = 15 μm, Ly1 = 40 μm, W = 5 μm. (**b**) Schematic of the hole array with geometrical parameters. Px2 = 15 μm, Py2 = 18 μm, Lx2 = 5 μm, Ly2 = 8 μm.

**Figure 4:**
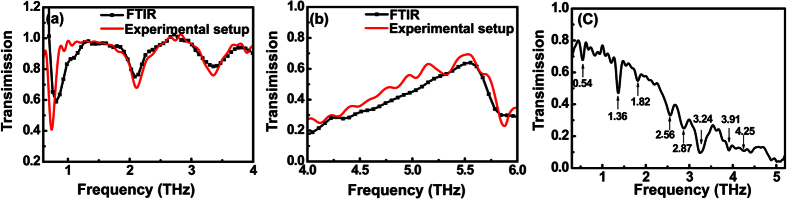
Transmissions of the two metamaterial samples and the lactose film. Frequency-dependent amplitude transmission of (**a**) the SRR and (**b**) the hole array samples. Red curves are the results measured by the proposed THz-TDS system while black curves are those measured by FTIR. (**c**) The amplitude transmission of the lactose film deposited on a silicon wafer. The absorption signature frequencies have been marked.

Though usually water vapor absorption lines are used to test the spectral accuracy of a THz-TDS, water vapor absorption under large humidity can not be clearly recognized at high frequencies due to the too weak THz spectral amplitude. So to verify the high frequency accuracy of this system, two metamaterial samples with a few designed frequency resonances were fabricated. One is a U-shape SRR array with typical LC and high-order plasmonic resonances. The other is a rectangular hole array with surface plasmonic polarization (SPP) resonance. These two 10 × 10 cm^2^-sized aluminum patterns were thermally evaporated on a 500-μm-thick semi-insulating silicon substrate. The unit cells of the SRR and the hole array structures with their geometrical parameters are shown in Fig. 3(a). In the experiment, the samples are placed midway between the two parabolic mirrors to ensure a nearly plane wave incidence. A piece of the blank silicon wafer, which is the same as the sample substrate, is used as the reference. The THz pulses propagate through the metamaterial samples at normal incidence with the same polarization of the electric field as the one in Fig. 3(a). The amplitude transmission t(ω) is defined as 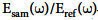, where 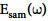 and 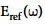 are the Fourier amplitudes of the transmitted THz fields through the sample and reference, respectively. In Fig. 4(a) the red line is the result measured with this system while the black line is obtained by a commercial FTIR system. The FTIR system, which has essentially different THz emission and detection mechanisms but covers nearly the whole THz band can objectively verify the spectral accuracy of our THz-TDS system. As shown in Fig. 4(a) there are three resonances at 0.74, 2.13 and 3.41 THz corresponding to three modes of the SRR sample: the LC, the second-order, and the third-order SPP resonances^38^. These resonances coincide with the results from the FTIR measurement by employing a commercial THz polarizer, considering the 0.06 THz frequency accuracy of the FTIR apparatus. Figure 4(b) shows the measured frequency-dependent amplitude transmission of the hole array sample. The resonance at 5.53 THz is primarily attributed to the resonant excitation of the SPP mode at the metal–dielectric interface. The measured result is also verified by the FTIR measurement. Besides the artificial metamaterials, pure lactose film on the semi-insulating silicon wafer was also measured in our system. The well-known absorption peaks of lactose at 0.54, 1.36, 1.82, 2.56, 2.89, 3.24, 3.91 and 4.25 THz are clearly observed as shown in Fig. 4(c), which coincides with the reported results in Refs [39,40]. It demonstrates that the DSTMS emission plus PC antenna detection scheme proposed here has an accurate frequency response and the frequency coverage up to 8 THz is experimentally reliable.

**Figure 5:**
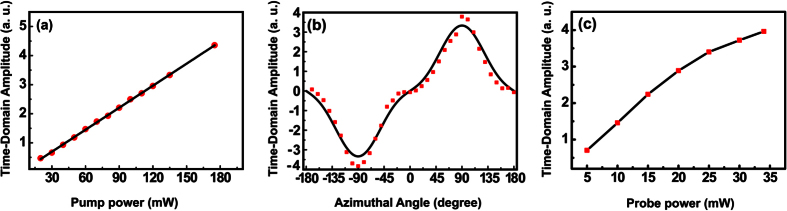
The time-domain amplitude dependences on the pump power, the azimuthal angle of the crystal and the excitation power on the PC antenna. (**a**) The THz amplitude increases linearly as the pump power rises. A linear fitting based on 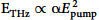 is shown in solid line, where α is a coefficient to simply contain all other factors that have no relation with the pump power. (**b**) The amplitude modulation when the crystal was rotated along its surface normal. Two maximum amplitudes are obtained at *θ* = −90°, 90°, where *θ* is the angle between b-axis and the polarization of the pump beam. (**c**) The THz time-domain amplitude increases linearly as probe power rises from 5 mW to 20 mW and turns to saturation when the probe power goes higher.

As mentioned above, the pump laser in our THz-TDS is a fiber femtosecond amplifier with small volume, low cost and fiber-coupling capability. But for feasible devices in realistic applications, a small pump power and low nonlinear effects in the fiber are of the same importance as the fiber integration capability. To investigate the pump power dependence of this system, we changed the power of the pump beam by two neutral density filters and measured the amplitude of the THz time-domain pulses, while the power of the probe beam remained at 25 mW. As shown in Fig. 5(a) the amplitude linearly increases by 8 times as the pump power rises from 20–180 mW, which is verified by linear fitting. The results are entirely in accordance with the second-order nonlinear feature of the OR process. Besides the pump power on the crystal, the obtained amplitude of the THz signal also changes when the DSTMS crystal is rotated along its normal, which originates from the polarization dependence of OR emission and antenna detection. Based on these two factors, we fit the azimuthal angle dependence of our system, as shown in Fig. 5(b), where the azimuthal angle *θ* was defined as the angle between the *b*-axis of the crystal (The pump beam incidents along the *c*-axis of the crystal onto its *ab* surface) and the polarization of the pump beam (the same direction with the dipole of the antenna). As we can see in Fig. 5(b), the fit is in good accordance with the experimental result. Two maximum amplitudes were obtained when the *a*-axis of the crystal is parallel to the polarization of the pump beam. Besides the pump power and the azimuthal angle dependences, the power of the probe beam also strongly influences the whole response of the THz-TDS system by determining the detection efficiency, which is investigated by changing the power of the probe beam from 5 mW to 35 mW (the breakdown limitation power) while keeping the pump power at 177 mW. Figure 5(c) demonstrates that the amplitude increases linearly as the probe power is raised from 5 mW to 20 mW. But after that, the amplitude turns to saturation when the probe beam power continues to rise^41^,^42^,^43^.

Even though in this work the PC antenna was still sampled by free space optics, its fiber coupling capability has been demonstrated by previous works^33^,^34^. The DSTMS emission plus PC antenna detection scheme proposed here has a promising future in developing compact ultra-broadband THz-TDS systems, which is urgently needed in many important areas. Firstly, both DSTMS and LTG InGaAs/InAlAs PC antenna have very good performances around 1550 nm, where pumping lasers, propagation devices and modulation components are relatively compact, flexible, low-costly and fiber-technique integratable, making the scheme much easier to be accepted by the society^33^,^34^. Secondly, the frequency coverage up to 8 THz is broader than most THz-TDS systems commercially available today, which means our proposal has a higher spectral signature identification capability. However, before the realistic application of this scheme, the remaining issues such as the echo from the substrate, the absorption of the InP substrate, the SNR of the whole system and the reason of the fine structures in the spectrum need more through studies.

## Conclusion

We presents a new scheme to generate and detect broadband THz wave. The proposed THz-TDS with a DSTMS crystal as the source and an LTG InGaAs/InAlAs PC antenna as the detector shows a frequency response up to 8 THz with a central frequency of 1.44 THz. The transmission of two metamaterial samples with different structures measured in this system coincides with the results measured by FTIR. And the absorption signatures of lactose tested in this system are in good accordance with the previously reported results. The performance dependence on the pump power, the azimuthal angle of the crystal and the probe excitation power is experimentally analyzed. This demonstration paves a way to realize a compact, low-cost and fiber technology friendly THz-TDS with a much broader frequency range in future.

## Additional Information

**How to cite this article**: Zhang, Y. *et al*. A Broadband THz-TDS System Based on DSTMS Emitter and LTG InGaAs/InAlAs Photoconductive Antenna Detector. *Sci. Rep*. **6**, 26949; doi: 10.1038/srep26949 (2016).
